# Leveraging endocrine sensitivity in ER-positive DCIS: Neoadjuvant therapy and nonoperative management

**DOI:** 10.1016/j.breast.2026.104898

**Published:** 2026-08-19

**Authors:** Ryoko Semba, Sarah Childs, Kate Saw, Davendra Segara, G Bruce Mann, Tadahiko Shien, Stephen Birrell, Yoshiya Horimoto, Goro Kutomi, Elgene Lim

**Affiliations:** a Garvan Institute of Medical Research, NSW, Australia; b Department of Breast Oncology, Juntendo University School of Medicine, Tokyo, Japan; c St Vincent's Hospital, Darlinghurst, NSW, Australia; d School of Clinical Medicine, Faculty of Medicine and Health, University of New South Wales, NSW, Australia; e The Royal Melbourne Hospital, Department of Surgery, University of Melbourne, VIC, Australia; f Department of Breast and Endocrine Surgery, Okayama University Hospital, Okayama, Japan; g Faculty of Health, University of Adelaide, South Australia, Australia; h Department of Breast Surgical Oncology, Tokyo Medical University, Tokyo, Japan

**Keywords:** Neoadjuvant endocrine therapy, Ductal carcinoma in situ, Risk stratification, Biomarkers

## Abstract

**Purpose:**

Ductal carcinoma in situ (DCIS) is a non-invasive precursor to breast cancer, frequently oestrogen receptor (ER)-positive and detected through screening. Neoadjuvant endocrine therapy (NET), established in ER-positive invasive breast cancer, is being investigated in DCIS both as a short-term biological response model and as a potential component of emerging non-surgical or active-surveillance strategies. This review synthesises current clinical and biological evidence on NET in ER-positive DCIS and identifies knowledge gaps and future directions.

**Methods:**

A systematic search (PubMed/MEDLINE, the Cochrane CENTRAL, Embase) was performed to identify prospective and retrospective studies evaluating NET in DCIS. Eligible studies included those assessing biological (Ki-67, PR expression) or radiological (lesion volume reduction) outcomes. Data were narratively synthesised due to study heterogeneity.

**Results:**

Four early-phase trials demonstrated consistent biological responsiveness of ER-positive DCIS to NET, with suppression of Ki-67 and PR expression, and occasional histologic regression. Aromatase inhibitors produced measurable radiologic downsizing in selected cohorts, although the clinical significance of these changes in DCIS remains uncertain. Pathologic complete response was uncommon, and a small proportion of cases were found to have occult invasive disease at surgery, underscoring the need for careful patient selection and cautious interpretation of early NET studies. Furthermore, the validity of short-term surrogate endpoints, such as Ki-67 suppression and radiological volume reduction, remains unproven regarding long-term prevention of invasive transformation.

**Conclusion:**

NET demonstrates consistent biological activity in ER-positive DCIS, supporting its use as a tool for tumour biology assessment rather than as a therapeutic alternative to surgery. Further prospective studies (COMET, LORETTA, RECAST, HORNEO01) are required to determine the safety, efficacy, appropriate patient population, and long-term outcomes of endocrine-inclusive personalised strategies. Given the exploratory nature and limitations of current data, these findings offer a conceptual framework that mandates robust, long-term prospective validation before any non-operative endocrine approach can be integrated into routine clinical practice.

## Highlights

• ER-positive DCIS shows a consistent biological response to neoadjuvant endocrine therapy.
• NET suppresses Ki-67 and PgR expression across early-phase clinical studies.
• Radiologic downsizing occurs in selected cohorts, though clinical significance remains uncertain.
• Pathologic complete response is uncommon, and occult invasion remains a key concern.
• NET is best utilized as a window-of-opportunity tool to inform risk stratification.

## Graphical abstract

**Figure:**
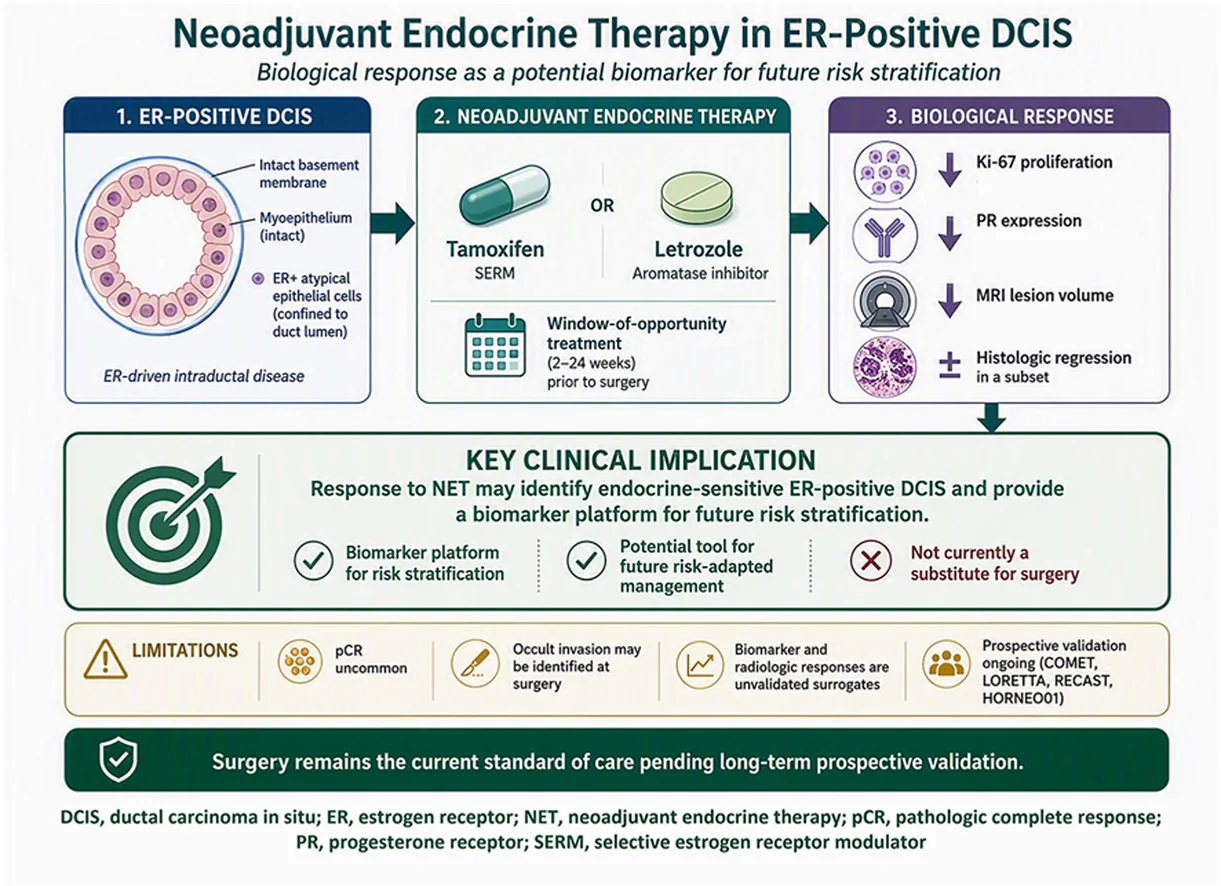

## 1 Background and rationale

Ductal carcinoma in situ (DCIS) is a pre-malignant breast condition characterised by the proliferation of atypical epithelial cells which remain confined within the milk ducts. DCIS is “non-invasive,” as these cells have not acquired the capacity to breach the myoepithelial layer or the underlying basement membrane and invade surrounding stromal tissue. While DCIS is widely regarded as a non-obligate precursor to invasive breast cancer (IBC), a substantial subset of lesions remain indolent and may never progress. Evidence from long-term observational cohorts suggests that 25–60% of untreated DCIS lesions progress to IBC during 9–24 years of follow-up, though these estimates are derived from small cohorts with inherent limitations [1]. Supporting its frequent subclinical presence, autopsy studies of women who died from unrelated causes have demonstrated occult DCIS in more than 10% of breast tissue samples [2]. In contrast, Lobular carcinoma in situ (LCIS) is biologically distinct from DCIS and primarily represents a risk marker rather than a direct precursor lesion [3].

DCIS represents a biologically heterogeneous group of lesions with molecular profiles largely paralleling those of IBC. Gene expression analyses have identified the same intrinsic subtypes, Luminal A, Luminal B, HER2-enriched, and Triple negative (Basal-like) in DCIS, suggesting that epithelial transformation and subtype differentiation occur early in tumourigenesis, whereas invasion represents a later event that develops only in a subset of women over their lifetime [4,5]. Using PAM50 gene expression analysis, Bergholtz et al. reported that DCIS comprised 43-55% luminal A, 8-20% luminal B, 14-25% HER2-enriched, and 11-22% basal-like subtypes [6,7]. Importantly, the HER2-enriched subtype has been shown to be strongly associated with the presence of an invasive component, suggesting that it may represent a biologically higher-risk entity with greater potential for progression compared with luminal lesions [8]. Interestingly, however, the overall proportion of HER2-positive lesions is higher in DCIS than in IBC, suggesting that many HER2-positive in situ lesions may not progress, possibly due to their higher immunogenicity or early detection bias [9,10].

Reflecting this heterogeneity, several ongoing therapeutic de-escalation studies, including the non-endocrine LORIS and LORD trials, have adopted molecular and pathological criteria to define “low-risk DCIS”, often characterised as screen-detected, non-high-grade (grade 1 or 2) lesions without comedo-necrosis and generally with hormone receptor–positive and HER2-negative status [[11], [12], [13]]. These features are hypothesised to enrich for biologically indolent disease and are considered the most plausible candidates for endocrine-based or active surveillance strategies, although their predictive validity for long-term outcomes remains incompletely established. Recent large-scale data highlight the inherent limitations of relying solely on conventional clinicopathological variables for risk stratification. In a comprehensive multinational pooled cohort study of over 47,000 women, Schmitz et al. demonstrated that while tumour size and margin status were statistically associated with ipsilateral breast events, the absolute risk differences were modest and the discriminatory ability of traditional clinicopathological factors alone was limited [14]. This underscores an urgent need for integrated approaches incorporating biological, imaging and functional response biomarkers to optimise patient selection for de-escalation.

The widespread adoption and improved sensitivity of mammographic screening has driven a marked increase in DCIS diagnoses, now accounting for 15-25% of all screen-detected breast lesions, compared to ∼2% prior to 1985 [15,16]. This sharp rise in DCIS diagnoses has not been accompanied by a decline in the number of IBC cases diagnosed at stage 3 or 4. This has raised discussion about the potential for overdiagnosis and overtreatment in a subset of screen-detected cases [17]. Current treatment algorithms for DCIS aim to prevent invasive transformation and mirror the management of low-to intermediate-risk IBC, typically involving surgery with or without adjuvant radiotherapy, followed by consideration of adjuvant endocrine therapy in oestrogen receptor (ER) -positive cases [18].

Current therapeutic approaches for DCIS can be associated with meaningful treatment-related morbidity. Surgery can result in chronic pain, altered body image, and psychosocial distress, while radiotherapy may cause local tissue fibrosis, skin changes, and, in rare cases, secondary malignancies. Adjuvant endocrine therapy often contributes to menopausal symptoms, sexual dysfunction, and cognitive effects. In this context, there is growing interest in treatment de-escalation across all modalities, including surgery, radiotherapy, and endocrine therapy. Tools such as DCISionRT [19,20] aim to personalise radiotherapy decisions by integrating molecular and clinical risk factors, supporting more tailored and potentially less intensive treatment strategies.

A key challenge is to better define the biological heterogeneity of DCIS and refine risk stratification to distinguish lesions with a low likelihood of progression from those at higher risk of invasive transformation. Improved risk stratification may ultimately facilitate more individualised treatment approaches, although robust biomarkers capable of guiding treatment de-escalation, including primary use of endocrine therapy or no active treatment, have yet to be established.

Approximately 80% of DCIS cases are ER-positive [21,22]. In this subset, adjuvant endocrine therapy has been shown to reduce both ipsilateral recurrence and contralateral new breast events, although its preventive effect may extend beyond the ER status of the index lesion. Large prevention trials, such as the IBIS-I study [23] and the more recent TAM-01 trial [24], have demonstrated that tamoxifen, at both standard and low doses, can significantly reduce the incidence of new breast cancer events, including DCIS in the preventive context. The TAM-01 trial demonstrated that low-dose Tamoxifen (5 mg daily) for three years reduced the risk of IBC by approximately 40% after 10 years of follow-up (annual rate per 1000 person-years, 11.3 with tamoxifen versus 19.5 with placebo; hazard ratio [HR], 0.58; 95% CI 0.35-0.95; p = 0.03) [24]. It was well tolerated at this dose, leading to increasing use of low-dose tamoxifen as adjuvant or preventive therapy for women following surgery for ER-positive DCIS. Given the demonstrated value of neoadjuvant endocrine therapy (NET) in assessing short-term biological response and prognostic markers in ER-positive IBC, illustrated by trials such as POETIC [25], its investigation in DCIS can be viewed as a parallel approach to explore endocrine responsiveness in a non-invasive setting, and as a logical extension of adjuvant prevention trials such as TAM-01 [24]. Unlike the adjuvant setting, the neoadjuvant approach permits paired pre- and post-treatment tissue sampling, allowing direct assessment of endocrine-induced biological changes within individual lesions. Among these biomarkers, short-term changes in Ki-67 have emerged as a well-established pharmacodynamic marker of endocrine response in IBC and represent a biologically plausible biomarker in DCIS. This provides a unique opportunity to evaluate biomarkers of endocrine sensitivity and resistance that may ultimately improve risk stratification. However, in DCIS, NET has been explored primarily as a biological response model rather than as a therapeutic alternative to surgery, and its clinical significance remains uncertain. Furthermore, the relevance of radiological downsizing or pathologic complete response in DCIS is unclear, as these endpoints have not been validated in non-invasive disease and may be confounded by biopsy-related tissue removal. Collectively, these limitations highlight a critical dissociation between endocrine-induced biological effects and validated clinical outcomes in DCIS, underscoring that surrogate response cannot currently be equated with clinical benefit. These considerations highlight the need for cautious interpretation of early NET studies, rigorous patient selection and long-term prospective follow-up in any future de-escalation strategies.

This review aims to synthesise current evidence for NET in ER-positive DCIS, outline its biological and clinical rationale, and discuss its potential role as a biomarker platform to inform future risk stratification and treatment de-escalation strategies.

## 2 Evidence for NET in early-stage invasive ER-positive breast cancer

Landmark trials such as P024 and IMPACT established the efficacy of aromatase inhibitors and tamoxifen as neoadjuvant endocrine therapies in ER-positive IBC, demonstrated the impact of endocrine therapy in tumour downsizing and increasing breast-conserving surgery rates in postmenopausal women [[26], [27], [28], [29]]. More recently, the POETIC trial, enrolling more than 4000 patients with ER-positive IBC, applied a perioperative design to evaluate changes in pre and post treatment Ki-67 in response to short-term 2-week NET [25]. Patients were stratified by Ki-67 dynamics into three prognostic groups: consistently low, suppressed, and persistently high. Outcomes were best in the consistently low group, intermediate in the suppressed group, and poorest in those with persistently high Ki-67, defined as >10% in this study. These findings established the prognostic value of short-term endocrine response, supporting the role of NET as a tool for biological risk stratification [25]. Similarly, the ACOSOG Z1031 trial evaluated three aromatase inhibitors in postmenopausal women with stage II–III ER-positive breast cancer, confirming comparable efficacy across agents [30]. By incorporating Ki-67 dynamics and the Preoperative Endocrine Prognostic Index (PEPI) score, the study demonstrated a possible role for NET in tumour downsizing to facilitate BCS and identify patients with excellent prognosis who may safely avoid chemotherapy [30].

The optimal duration of NET in early-stage IBC is highly variable and depends on its intended clinical objective. Short-term NET (typically 2–4 weeks) is used to assess biological response using biomarkers such as Ki-67, as in POETIC and IMPACT [25,[27], [28], [29]]. On-treatment Ki-67 cut-offs derived from invasive-cancer NET programmes provide a pragmatic reference for DCIS research; notably, residual Ki-67 ≤ 10% (or alternatively a relative reduction, e.g. ≥50%) has been used to define endocrine responsiveness in trials such as POETIC and ACOSOG Z1031 [30,31]. In contrast, longer treatment durations (12–24 weeks) are generally required to achieve meaningful tumour downsizing or to improve BCS rates, as demonstrated in P024 and Z1031 [26,30]. Collectively, these findings suggest that NET represents a useful approach for assessing tumour biology and optimising surgical decision-making in early-stage ER-positive breast cancer, providing a rationale for exploring its potential application in DCIS.

## 3 Materials and methods

This systematic review was conducted in accordance with the Meta-analysis of Observational Studies in Epidemiology (MOOSE) guideline [32]. The review aimed to synthesise clinical and biological evidence from published observational and interventional studies investigating NET in ER-positive DCIS of the breast. The review protocol was not prospectively registered, as no quantitative meta-analysis was planned.

### 3.1 Data sources and search strategy

A comprehensive literature search was performed using PubMed/MEDLINE, Embase, and the Cochrane Central Register of Controlled Trials (CENTRAL) to identify studies investigating NET in patients with ER-positive DCIS. Clinical trial registries (ClinicalTrials.gov and Australia New Zealand Clinical Trials Registry [ANZCTR]) and reference lists of relevant reviews were also screened to identify additional eligible studies. The following keywords and Boolean operators were applied.

• **Pubmed/MEDLINE**

Ductal carcinoma in situ OR DCIS AND neoadjuvant endocrine therapy OR preoperative endocrine therapy OR tamoxifen OR aromatase inhibitor OR letrozole OR anastrozole OR fulvestrant OR SERD AND breast.

• **Cochrane CENTRAL**

Ductal carcinoma in situ OR DCIS AND neoadjuvant endocrine therapy OR preoperative endocrine therapy OR tamoxifen OR letrozole OR anastrozole OR fulvestrant OR SERD.

• **Embase**

Ductal carcinoma in situ OR DCIS AND neoadjuvant endocrine therapy OR preoperative endocrine therapy OR tamoxifen OR aromatase inhibitor OR letrozole OR anastrozole OR fulvestrant OR SERD AND breast, with Emtree terms applied where appropriate.

Searches were last updated in July 2025, and reference lists of relevant reviews and included studies were also manually screened to identify any additional eligible publications.

## 4 Eligibility criteria

Studies were considered eligible for inclusion if they met the following criteria.

1. **Population**: Patients diagnosed with DCIS of the breast.
2. **Intervention**: Neoadjuvant or preoperative endocrine therapy with tamoxifen, aromatase inhibitors, fulvestrant, or investigational hormonal agents.
3. **Design**: Prospective trials, retrospective cohort studies, or randomised controlled trials (RCTs).
4. **Outcomes**: Studies reporting at least one of the following:
  o Change in Ki-67 or other biomarkers
  o Tumour size reduction on imaging or pathology
  o Incidence of invasive cancer at surgery or follow-up
5. **Language**: Articles published in English.

## 5 Exclusion criteria

Studies were excluded if they met any of the following.

1. Case reports, editorials, review articles, or abstracts without full-text availability.
2. Preoperative treatment duration less than 2 weeks, unless biological response was reported in sufficient detail.
3. Studies in which DCIS data could not be separated from invasive disease or lacked clarity on treatment duration and outcomes.
4. Non-English publications.

### 5.1 Study selection and data extraction

Two reviewers independently screened titles and abstracts to identify potentially relevant articles. Full texts of selected studies were assessed for eligibility. Discrepancies were resolved by discussion with a third reviewer.

Data were extracted using a predefined template including.

1. Study characteristics (author, year, country, design)
2. Patient characteristics (age, ER status, sample size)
3. Type, dose and duration of endocrine therapy
4. Primary outcomes (biomarker changes, radiological/pathological response, invasive cancer incidence)

**Fig. 1:**
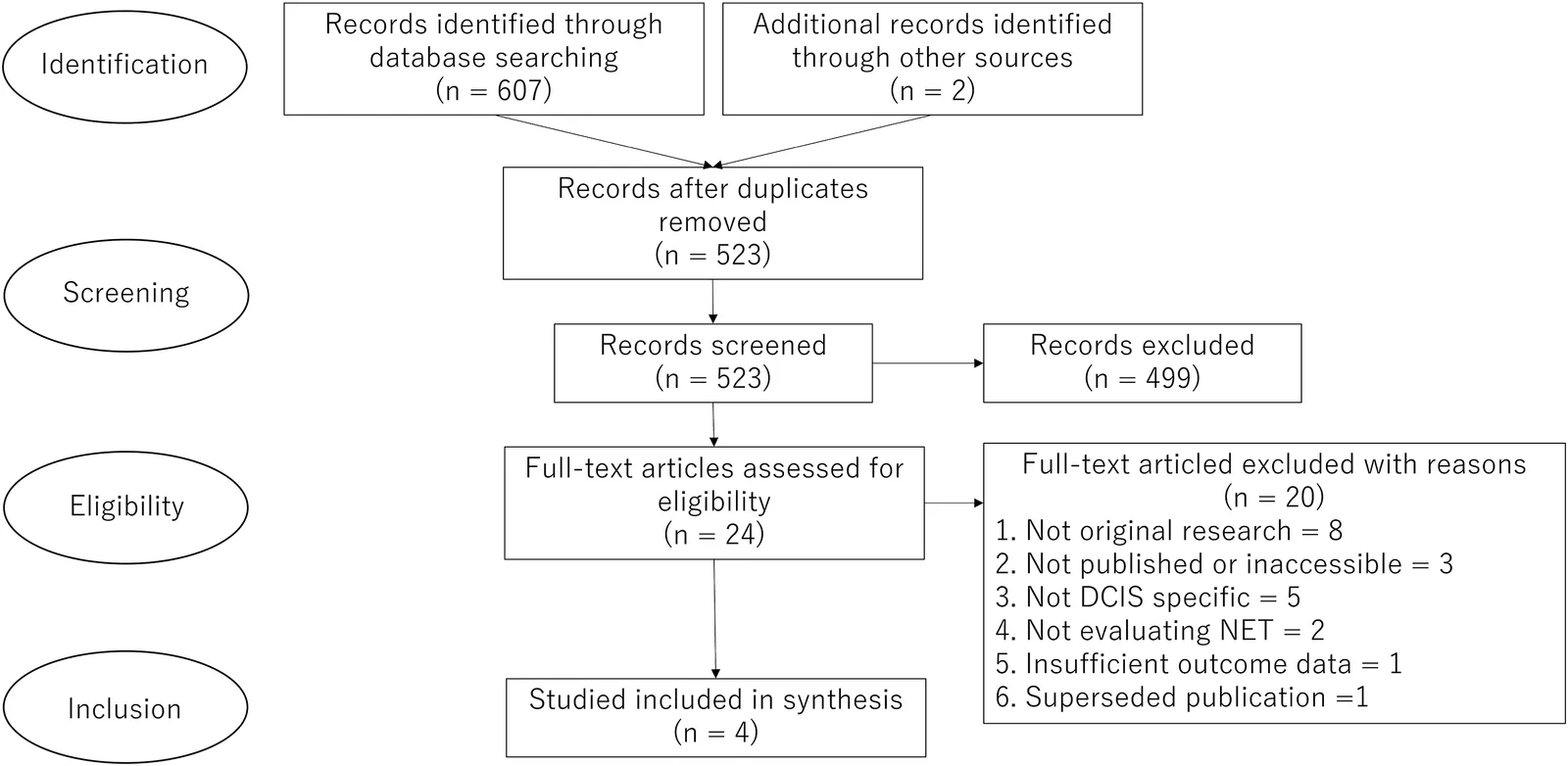
Flow Diagram of Study SelectionFlow diagram illustrating the identification, screening, eligibility assessment, and inclusion of studies evaluating neoadjuvant endocrine therapy in ductal carcinoma in situ.

A total of 607 records were identified through database searches and 2 through other sources; after screening and eligibility assessment, 4 studies were included in the final synthesis (Table 1, Fig. 1). A formal risk-of-bias assessment using standardised quality scoring tools was not performed as the review included only four highly heterogenous early-phase trials with substantially different designs, interventions, and endpoints, limiting the interpretability and comparability of standard risk-of-bias tools. Consequently, a descriptive critical appraisal of each trial's biological and clinical context was deemed more appropriate for the exploratory nature of the available evidence.

**Table 1:** Trials evaluating neoadjuvant endocrine therapy in ER-positive DCIS.

Study (Author, Year)	Design	n	Population	Intervention (daily dose)	Duration	Mean Ki67 change from baseline	Radiological response from baseline	Invasive upstaging at surgery, n/N (%)	pCR (no residual DCIS), n/N (%)
**Khan et al., 2023** [33]	Phase II, randomised	48	Pre- and postmenopausal	Tamoxifen 20 mg	4–10 weeks	10.2% → 5.3% (p < 0.001)	NA	8/48 (16.7%)	8/48 (16.7%)
		42		4-OHT gel 4 mg		8.8% → 7.8% (p = 0.2)		3/42 (7.1%)	7/42 (16.7%)
**CALGB 40903 (Hwang), 2020** [34]	Phase II, single-arm	79	Postmenopausal	Letrozole 2.5 mg	6 months	12.0% → 6.3% (p = 0.007)	↓ 71.7% MRI volume at 6 months, ↓ 61% at 3 months	6/59 (10.2%)	9/59 (15.3%)
**Chen et al., 2009** [35]	Non-randomised	9	Premenopausal	Tamoxifen 20 mg	3 months	7.5% (p = 0.037)	NA	0	0
		14	Postmenopausal	Letrozole 2.5 mg		14.4% (p = 0.013)		2/14 (14.3%)	2/14 (14.3%)
**Takagi et al., 2013** [36]	Single arm	10	Postmenopausal	Letrozole 2.5 mg	3–27 weeks	10% → 3% (p = 0.005)	Mean 11 → 9 mm on US (p = 0.17)	3/10 (30%)	0/10 (0%)

Abbreviations: 4-OHT, 4-hydroxytamoxifen; AE, adverse event; AI, aromatase inhibitor; DCIS, ductal carcinoma in situ; MRI, magnetic resonance imaging; NA, not available; pCR, pathological complete response; PR, progesterone receptor; US, ultrasound.

## 6 Data synthesis

Based on these methodological considerations and the heterogeneity of available studies, a narrative synthesis was performed. A descriptive summary of each study was presented in tabular form, and key outcomes were compared across trials. Meta-analysis was not conducted due to variability in endpoints and study designs.

## 7 Clinical trials of NET in DCIS

Several studies have explored NET in DCIS, summarised in Table 1. These collectively demonstrate that ER-positive lesions are biologically responsive to endocrine manipulation. The largest of these was a randomised clinical trial comparing the efficacy and tolerability of 4-10 weeks of preoperative oral tamoxifen compared to transdermal 4-hydroxytamoxifen (4-OHT) gel in 90 women with ER-positive DCIS [33]. Oral tamoxifen demonstrated superior biological activity, achieving a significant reduction in median Ki-67 (10.2 vs 5.3%, p < 0.001) and higher intra-tumoural concentrations of active metabolites such as endoxifen. In contrast, the transdermal 4-OHT gel failed to reach comparable tissue levels and did not produce a meaningful suppression of proliferation markers, falling short of non-inferiority. Although the transdermal formulation was better tolerated with fewer systemic side effects, its limited efficacy underscored the continued clinical relevance of standard oral tamoxifen for reliable endocrine response.

The phase II CALGB 40903 (Alliance) trial enrolled 79 postmenopausal women with ER-positive DCIS to receive 6 months of preoperative Letrozole 2.5 mg daily [34]. MRI assessments demonstrated a substantial reduction in lesion volume (median reductions 60-70%). Biological efficacy was demonstrated through immunohistochemical assessment of declines in ER, PR, and Ki-67 expression. At surgery, 15% of patients achieved a pathologic complete response (pCR), while 10% were found to have IBC, which were likely occult and undiagnosed at the time of DCIS. These findings confirm that neoadjuvant aromatase inhibition can induce measurable biological and imaging responses in ER-positive DCIS, with some patients obtaining a pCR.

Two smaller single-institution studies reported similar results. Chen et al. enrolled 23 women with ER-positive DCIS to receive ∼3 months of NET, tamoxifen 20 mg daily for premenopausal and Letrozole 2.5 mg daily for postmenopausal women [35]. Paired biopsy samples, compared to untreated controls, were assessed for pathological and biological effects, including changes in ER, PR, Ki-67, CD68 (macrophage infiltration), and caspase-3 (a marker of apoptosis). NET led to significant reductions in PR and Ki-67 expression, particularly in the letrozole group, where the mean Ki-67 decrease was 14.4% (p = 0.013), compared to 7.5% in the tamoxifen group (p = 0.037). An increase in CD68-positive macrophages suggested activation of local inflammatory responses, while caspase-3 levels remained unchanged. Similar to the larger studies, two cases demonstrated complete pathological response, with only atypical ductal hyperplasia (ADH) at surgery. Takagi et al. investigated intratumoural estrogen dynamics following neoadjuvant letrozole treatment for 3-27 weeks in 10 post-menopausal women with ER-positive DCIS. [36]. Immunohistochemical analysis revealed marked suppression of the median Ki-67 expression from 10 to 3% (p = 0.005) and median progesterone receptor (PR) expression (% of positive tumour cells) from 93 to 18% (p = 0.018). Expression of tumoural estrogen-responsive genes was downregulated in 92–97% of cases, and longer treatment durations (14-27 weeks) were associated with a decrease in ultrasound diameter (from 11 to 9 mm, p = 0.043). These studies confirmed that preoperative aromatase inhibition effectively suppressed tumoural estrogen signalling and proliferation in situ.

Collectively, these four studies provide consistent evidence that ER-positive DCIS is biologically responsive to endocrine manipulation. Across heterogeneous study designs, both short- and longer-duration aromatase inhibitors and tamoxifen reproducibly suppressed proliferative activity (Ki-67) and ER signalling, with aromatase inhibitors demonstrating potential for radiological downsizing with longer duration therapy [34]. These biomarker and imaging signals indicate that NET exerts meaningful on-target effects in situ and can, in some cases, produce histologic regression (occasional conversion to atypia/benign histology) or pCR in a minority of patients.

Several caveats temper enthusiasm about routine clinical adoption of NET as a non-surgical strategy for ER–positive DCIS. First, rates of pCR were, as expected, low, indicating that a limited duration of endocrine therapy alone is unlikely to eradicate most lesions. In these studies, pCR generally referred to the absence of residual DCIS (“no residual DCIS”) at surgery; however, in some cases, diagnostic procedures such as vacuum-assisted or surgical biopsy may have removed a substantial proportion of the lesion, resulting in an apparent rather than true pathological response. Second, a non-negligible proportion of patients experienced progression or were found to have IBC during observation or at the time of surgery. This likely reflects the presence of occult IBC not detected on the initial diagnostic biopsy and underscores the importance of careful patient selection, rigorous baseline imaging, and close follow-up in any strategy that defers definitive surgical management. Finally, interpretation of the existing literature is constrained by marked heterogeneity in trial design—including endocrine agent, dose, route, and duration—as well as small cohort sizes and variable primary endpoints (biomarker modulation, radiological change, or pathological outcome). Crucially, a major methodological limitation is the unproven validity of surrogate endpoints, such as Ki-67 suppression and radiological volume reduction, in the context of non-invasive disease. While short-term Ki-67 down-regulation reflects an immediate biological anti-proliferative response to estrogen deprivation, it remains entirely unknown whether this transient biomarker modulation translates into the long-term prevention of invasive transformation or improved breast cancer-free survival. Furthermore, there are substantial pre-analytical, analytical, and interpretive variability with Ki67. The International Ki67 Working Group has shown poor reproducibility across laboratories, particularly in the clinically critical intermediate range (∼5–30%), limiting its use as a standalone biomarker for treatment decisions [37]. Similarly, radiological downsizing on MRI or ultrasound can be highly confounded by biopsy-related tissue removal or patchy, non-uniform regression of DCIS lesions, making imaging a potentially unreliable indicator of true pathological clearance. Until these surrogate markers are rigorously correlated with long-term oncologic outcomes in large prospective cohorts, their use remains strictly experimental and insufficient to guide routine clinical decision-making. In addition, variability in biomarker assessment, particularly for Ki-67, further limits cross-study comparability and precludes firm generalisation of these findings.

During the COVID-19 pandemic, the value of NET was further highlighted when elective breast surgeries were widely postponed. In a large National Cancer Database analysis including 109,990 women with hormone receptor–positive DCIS, Williams et al. identified 276 patients (0.3%) who received NET prior to surgery. The mean duration of NET was 74 days, and the median time to surgery was significantly longer in the NET cohort than in the no-NET cohort (87.5 vs 35 days, p < 0.001). Despite this delay, the incidence of IBC at surgery was similar between the two groups (15.6 vs 12.3%, p = 0.10) [38]. This real-world experience suggests that short-term NET may serve as a reasonable bridging or alternative strategy for selected patients with DCIS when surgery is delayed or deferred.

Finally, Glencer et al. (2022) reported 71 patients with ER-positive DCIS who declined immediate surgery; approximately 89% accepted primary endocrine therapy and were managed with active surveillance [39]. After a mean follow-up of 8.5 years, 52% of patients remained on active surveillance without developing IBC. Of the remaining 48% of patients, 34 underwent surgery due to clinical or radiological suspicion of progression during active surveillance; 19 (56%) were found to have IBC, while 15 (44%) had residual DCIS. Most progression events occurred within the first four years of follow-up. Interestingly, progression risk appeared to vary according to MRI-based features, including background parenchymal enhancement (BPE), which reflects the response of normal breast tissue to contrast and is associated with hormonal activity and tissue density. In this cohort, BPE ranged from 8.7% in the low-risk group, 20.0% in the intermediate-risk group, and 68.2% in the high-risk group. These observations suggest a potential exploratory role for MRI and functional imaging markers in refining patient selection for non-surgical management, but their predictive value in DCIS remains unproven and requires prospective validation. Notably, prior studies in IBC have shown that higher BPE is associated with worse invasive disease-free survival and that reductions in BPE during neoadjuvant chemotherapy may correlate with better pathological response; however, these findings cannot be directly extrapolated to DCIS [40].

The optimal duration of endocrine therapy for DCIS remains to be defined and may depend on its intended clinical purpose. From a translational perspective, the recurring biomarker patterns support the use of short “window-of-opportunity” courses (typically 2–4 weeks) to assess endocrine responsiveness. In contrast, longer treatment durations of several months may be appropriate when the objective is to achieve a measurable reduction in disease extent or imaging evidence of lesion shrinkage, thereby potentially improving surgical planning and breast-conserving options. Overall, the ideal treatment length is likely to vary according to the balance between biological assessment, therapeutic intent, and tolerability, and should be refined through future prospective studies.

Tolerability is an important clinical issue in the use of endocrine therapy for DCIS. Tamoxifen and aromatase inhibitors share the typical adverse event profile observed in IBC, including vasomotor symptoms, an increased risk of thromboembolic events and endometrial carcinoma with tamoxifen 20 mg, and bone density loss and arthralgia with aromatase inhibitors. A better-tolerated endocrine therapy may potentially increase the clinical acceptability of primary or NET for the management of ER + DCIS. The TAM-01 trial reported similar patient-reported outcomes with tamoxifen 5 mg compared with placebo, except for a slight increase in hot flushes, with an incidence ratio of 1.46 (95% CI 1.05-2.00), and no difference in serious adverse events [24]. Considering its efficacy in secondary prevention following a diagnosis of ER-positive DCIS, it would be logical to evaluate this well-tolerated dose in the preoperative setting.

Future research should first clarify the intended clinical role of NET in ER-positive DCIS, not as mutually exclusive options, but potentially serving both as a short-term tool to assess endocrine responsiveness and tolerance, and as a longer-term therapeutic strategy to induce tumour regression and inform decisions regarding surgery or adjuvant radiotherapy. For NET as a biological response assay, adequately powered prospective studies are needed to validate early biomarker changes, such as Ki-67 suppression and hormone receptor modulation, and/or imaging response biomarkers such as MRI or contrast-enhanced mammography, as reliable predictors of long-term outcomes. Ultimately, the clinical value of these biomarkers will depend not simply on demonstrating endocrine responsiveness but on their ability to predict long-term oncologic outcomes. An ideal biomarker should reliably distinguish lesions destined to remain indolent from those likely to progress to invasive disease, demonstrate reproducibility across laboratories and imaging platforms, and provide prognostic information beyond conventional clinicopathological factors. Whether currently available biomarkers fulfil these requirements remains unknown. Standardising short-duration protocols will be essential to ensure reproducibility and clinical utility. For NET as a therapeutic approach, future trials should investigate its capacity to induce histologic regression or radiologic downsizing in carefully selected patients. Integration of imaging biomarkers, including BPE, may improve risk stratification and help identify candidates for non-surgical management. Response-adaptive trial designs incorporating serial imaging and tissue sampling could help define thresholds for safely deferring surgery. Finally, comparative studies evaluating different endocrine agents, formulations, and treatment durations will be critical to optimise efficacy, tolerability, and patient adherence across diverse populations.

## 8 Primary endocrine therapy for DCIS

Ongoing large trials incorporating primary endocrine therapy and/or surveillance for DCIS are summarised in Table 2, and we await long-term results from these studies. As opposed to the NET trials, such an approach does not necessarily have surgery as an end point, and the optional duration of endocrine therapy has not been determined. An important limitation when interpreting non-operative or NET studies in DCIS is the inherent diagnostic uncertainty of preoperative biopsy. Occult invasive disease may be missed at initial sampling, leading to apparent rather than true progression during observation. Similarly, the histopathological distinction between DCIS and ADH can be challenging, potentially contributing to variability in reported response or progression rates. Recently, several de-escalation trials have begun to investigate whether selected patients with low-risk, ER-positive DCIS can be safely managed without immediate surgery, using active monitoring with or without endocrine therapy [41]. These studies have renewed interest in NET as a potential component of non-operative strategies. While these studies demonstrate the impact of endocrine therapy in ER-positive DCIS, what is currently lacking is the utility of the response as a prognostic or predictive biomarker.

**Table 2:** Trials of primary endocrine therapy and/or active surveillance for DCIS.

Trial	Registry ID	n	Design/intervention	ET duration	Primary endpoint	Follow-up	Status (as of 2026)
**COMET**	NCT02926911	997 (actual)	Randomised: active monitoring ± ET vs guideline-concordant care (surgery ± RT ± ET)	Per treating clinician	Ipsilateral invasive breast cancer rate at 2 years	To 2028	Active, not recruiting
**LORETTA**	JCOG1505	340 (actual)	Tamoxifen (surgery deferred)	5 years	Ipsilateral invasive breast cancer-free survival	Long-term follow-up ongoing	Completed; primary results reported
**RECAST**	NCT06075953	400 (target)	Tamoxifen, AI, elacestrant, endoxifen, or testosterone + AI, followed by active surveillance	6 months	Proportion remaining on active surveillance at 7 months	Up to 5 years	Recruiting
**HORNEO01**	NCT04666961	262 (target)	Tamoxifen (premenopausal) or anastrozole (postmenopausal)	6 months	Proportion of conservative surgeries after ET	10 years	Recruiting

Abbreviations: AI, aromatase inhibitor; DCIS, ductal carcinoma in situ; ER, estrogen receptor; ET, endocrine therapy; NET, neoadjuvant endocrine therapy; RT, radiotherapy.

To critically appraise the non-operative strategies summarised in Tables 2 and it is essential to contextualize them against other landmark active surveillance trials that omit hormonal intervention entirely, thereby highlighting the ongoing uncertainty regarding the absolute added value of endocrine therapy. For instance, while the Japanese LORETTA trial mandated endocrine therapy and the US COMET trial permitted it, the European LORD trial and the UK LORIS trial allocated patients with low-risk DCIS to active monitoring without any endocrine treatment. This fundamental divergence in trial designs underscores that endocrine treatment is not universally considered an essential backbone of non-surgical management [[11], [12], [13]]. Rather, it reflects a continuous debate over whether active surveillance alone is sufficient for carefully selected, indolent lesions, or if concurrent systemic endocrine suppression is required to safely prevent invasive transformation.

The COMET trial enrolled women with low-risk, HR-positive DCIS and randomised patients to active monitoring, with or without endocrine therapy, versus standard care (surgery ± radiotherapy) [41]. At two years, the incidence of IBC was 4.2% in the active monitoring arm and 5.9% in the standard care arm (HR 0.8, 95% CI 0.3–2.3; p = 0.73), indicating comparable short-term outcomes. However, it is important to note that the slightly higher incidence of IBC in the surgical arm likely reflects incidental detection at the time of surgery rather than true biological progression. Additionally, only approximately 70% of participants in the active-monitoring arm received endocrine therapy, which may complicate the interpretation of long-term outcomes. Longer-term follow-up is ongoing to determine whether these outcomes are maintained over time, which will be critical for defining the safety and durability of non-surgical management approaches.

The LORETTA trial is a single-arm phase III Japanese multicentre study evaluating active surveillance combined with mandatory endocrine therapy in women aged 40 years and older diagnosed with ER-positive DCIS [42]. The primary endpoint was the 5-year cumulative incidence of ipsilateral invasive breast cancer (CIPIC), with a prespecified safety threshold requiring the upper bound of the 95% confidence interval to remain below 7%. Following interim analyses, the independent data and safety monitoring committee recommended early termination after 18 invasive events were observed, corresponding to an estimated 5-year CIPIC of 9.8% (95% CI, 5.2–16.1) at a median follow-up of 36 months. Subgroup analyses identified imaging-defined tumour size as the principal determinant of invasive progression, with tumors ≥2 cm associated with higher risk on mammography and ultrasound, and a borderline association on MRI. No significant associations were observed with nuclear grade, HER2 status, progesterone receptor expression, or age. Together, these findings highlight the central role of tumour size in defining “low-risk” DCIS and suggest that more restrictive size thresholds may be required when considering non-surgical endocrine-based management. As these data were reported at the 2025 San Antonio Breast Cancer Symposium and are not yet peer reviewed, they should be interpreted with appropriate caution [43].

The RECAST trial is a phase II platform trial testing standard and investigational endocrine agents (e.g., tamoxifen, aromatase inhibitors, elacestrant, endoxifen, testosterone + anastrozole) in ER-positive DCIS. Patients receive 6 months of endocrine therapy with MRI-based response assessment. The primary endpoint is the proportion of patients eligible for continued active surveillance at 7 months. Finally, HORNEO01 is a phase II French trial evaluating neoadjuvant tamoxifen or anastrozole in patients with extensive ER-positive DCIS initially indicated for mastectomy. The study aims to increase breast-conserving surgery rates by assessing MRI-based treatment response after 6 months of hormone therapy.

Beyond conventional clinicopathological stratification based on tumour size, nuclear grade, and ER status, emerging molecular pathology data suggest that additional biological factors may further refine risk assessment in DCIS. Notably, PIK3CA mutations are detected in approximately 30% of DCIS cases and are associated with constitutive activation of the PI3K–AKT–mTOR pathway [44]. In this context, tumour proliferation may become less dependent on estrogen receptor signalling, potentially conferring relative resistance to selective estrogen receptor modulators. These observations raise the possibility that molecular features such as PIK3CA mutation status could influence responsiveness to endocrine-based non-surgical strategies and may warrant consideration in future trial design and patient selection.

## 9 Conclusions

NET has demonstrated biological activity in ER-positive DCIS, capable of inducing measurable reductions in proliferation (Ki-67) and PR expression, radiologic tumour downsizing, and, in some cases, histologic regression. Across heterogeneous early-phase trials, both tamoxifen and aromatase inhibitors demonstrate consistent endocrine responsiveness, supporting their utility as a “window-of-opportunity” model to probe tumour biology and refine prognostic stratification. However, despite these biological effects, pathologic complete responses remain uncommon, and a subset of patients harbour occult or progressive invasive disease, emphasising the necessity for appropriate risk-stratified clinical translation.

Future research efforts should focus on validating biological and imaging biomarkers, including dynamic Ki-67 suppression or changes in PR expression, which may serve as functional biomarkers of ER pathway activity which have been linked to endocrine sensitivity and resistance, and MRI-based features such as size and BPE. Integrating these markers, alongside standard clinicopathological factors may enable the development of composite risk models that can identify patients who could be candidates for prospective evaluation of treatment de-escalation strategies.

Ongoing prospective trials may clarify the evidence on clinical safety, durability, and optimal parameters for incorporating NET into personalised, less invasive management strategies for ER-positive DCIS [41,42]. While NET may eventually signal a shift in how DCIS is managed, supporting a move away from uniform surgical approaches toward a more biologically informed, precision-focused strategy that considers endocrine sensitivity in ER-positive disease and incorporates risk-stratified management, the current body of evidence remains strictly exploratory. Such an approach has the potential to reduce overtreatment, improve patient quality of life, and better align therapeutic decisions with underlying oncologic risk, although robust prospective validation with long-term oncologic outcomes is absolutely required before any shift in routine clinical practice can be recommended.

## CRediT authorship contribution statement

**Ryoko Semba:** Conceptualization, Investigation, Methodology, Project administration, Visualization, Writing – original draft, Writing – review & editing. **Sarah Childs:** Conceptualization, Writing – review & editing. **Kate Saw:** Conceptualization, Writing – review & editing. **Davendra Segara:** Writing – review & editing. **G Bruce Mann:** Writing – review & editing. **Tadahiko Shien:** Writing – review & editing. **Stephen Birrell:** Writing – review & editing. **Yoshiya Horimoto:** Writing – review & editing. **Goro Kutomi:** Writing – review & editing. **Elgene Lim:** Conceptualization, Supervision, Writing – review & editing.

## Disclosure statement

Stephen Birrell is a director or advisor to entities connected with the testosterone + aromatase inhibitor programme referenced in RECAST trial (Clinicaltrials.gov identifier NCT 06075953) and is a signatory to related licence agreements between HAVAH Therapeutics (licensor) and Wellend (licensee). Relevant agreements and the contemplated transfer of the licence are documented in the company records. All other authors declare no competing interests.
